# The complete mitochondrial genome of ﬂyingfish (*Cheilopogon spilonotopterus*) from South China Sea

**DOI:** 10.1080/23802359.2018.1481787

**Published:** 2018-06-18

**Authors:** Lei Xu, Xuehui Wang, Hong Li, Feiyan Du

**Affiliations:** a South China Sea Fisheries Research Institute, Chinese Academy of Fishery Sciences, Shanghai, China;; b Guangdong Provincial Key Laboratory of Fishery Ecology and Environment, Guangdong, China;; c Key Laboratory of South China Sea Fishery Resources Development and Utilization, Ministry of Agriculture, Guangzhou, China

**Keywords:** Mitochondrial genome, Cheilopogon spilonotopterus, South China Sea

## Abstract

Flyingfishes are epipelagic specialists that are easily distinguished by their enlarged fins, which are used for gliding leaps over the surface of the water. In this study, we described the complete mitochondrial genome of *Cheilopogon spilonotopterus*. The genome is 16,527 bp in length, encoding the standard set of 13 protein-coding genes, 22 tRNA genes and two rRNA genes, with circular organization. The overall base composition of the whole mitochondrial genome was A (28.86%), T (26.96%), G (16.66%) and C (27.52%) with an AT bias of 55.82%. The ATG initiation codon is used in all protein-coding genes except *COX1*, and the stop codons of all the 13 protein-coding genes were complete.

The ﬂyingfish family Exocoetidae includes approximately 50 species that are distributed across the tropical and subtropical regions of the Pacific, Atlantic, and Indian oceans. Fyingfishes as a key element in epipelagic food webs, they feed on zooplankton and transfer energy from lower levels of the trophic system to top predators. The most particular morphological character of flyingfish is its exceptionally large pectoral fins (Davenport 1994). The genus *Cheilopogon*, with most species and morphologically variable within flyingfishes, is not monophyletic (Lewallen et al. 2011). Recently, the complete mitogenomes of three flyingfishes in *Cheilopogon* have been reported (Chou et al. 2016). Here, we sequenced and annotate mitogenome of *Cheilopogon spilonotopterus* to provide molecular information for genetically understanding of flyingfishes.

Whole genomic DNA was extracted from muscle tissue of one specimen of *C*. *spilonotopterus* collected from the South China Sea (12°30′N, 114°30′E) using TIANamp Marine Animals DNA Kit (TIANGEN, Beijing, China). The concentration for use as a PCR template was adjusted to an A _260_ of about 0.05–0.2. The collected specimen and extracted DNA were stored in Guangdong Provincial Key Laboratory of Fishery Ecology and Environment. The complete mitochondrial genomes of *C*. *spilonotopterus* was sequenced using PCR primers designed from highly conserved regions of transfer RNA (tRNA) sequences of related species (Chou et al. 2016) with additional specific primers designed as required from sequences already obtained. Long-PCR amplifications were performed by thermo-cycling using five pairs of primers and PCR amplicons were subjected to build up genomic library and pair-end sequencing by MiSeq. The COI sequence of *C*. *spilonotopterus* was used as reference seeds for iterative assembly by MITObim v.1.8 (Hahn et al. 2013). SeqMan v.7.1.0 was used for the mitogenome assembly and annotation (Swindell and Plasterer 1997). Transfer RNA genes were predicted using online software tRNAScan-SE 1.21 (Lowe and Eddy 1997). All Protein coding gene (PCGs) are aligned independently, then concatenated to be applied for phylogenetic reconstruction with other cephalopods in MrBayes v 3.12 (Ronquist and Huelsenbeck 2003) using relaxed clock model.

The *C*. *spilonotopterus* mitochondrial genome forms a 16,527 bp closed loop (GenBank accession number MH251624). The overall base composition of the whole mitochondrial genome was A (28.86%), T (26.96%), G (16.66%) and C (27.52%) with an AT bias of 55.82%. This mitochondrial genome represents a typical *Cheilopogon* mitochondrial genome and matches with the *C. unicolor* genome, in which it comprises 13 protein-coding gene, 22 transfer RNA genes and two rRNA genes (12S rRNA and 16S rRNA) and one A + T-rich region which could also be termed as control region. The ATG initiation codon is used in all protein-coding genes except *COX1* (GTG), and the stop codons of all the 13 protein-coding genes were complete. Meanwhile, the longest protein-coding genes of these species was *ND5* (1836 bp), whereas the shortest *ATP8* (162 bp). *lrRNA* and *srRNA* genes are 1685 bp and 943 bp in length separately, and the length of D-loop is 1010 bp. All the 22 typical tRNAs possess a complete clover leaf secondary structure, ranging from 68 bp to 73 bp. The Bayesian inference phylogenetic tree showed that *C*. *spilonotopterus* firstly grouped with species of *C.atrisignis* and *C. cyanopterus* (Figure 1). Phylogenetic tree indicates that *Cheilopogon* is not monophyly. We have the confidence to construct phylogenetic trees, based on the complete the mitochondrial genomes, but the evolution history of flying fishes still needs future research to be clearly resolved.

**Figure 1.**
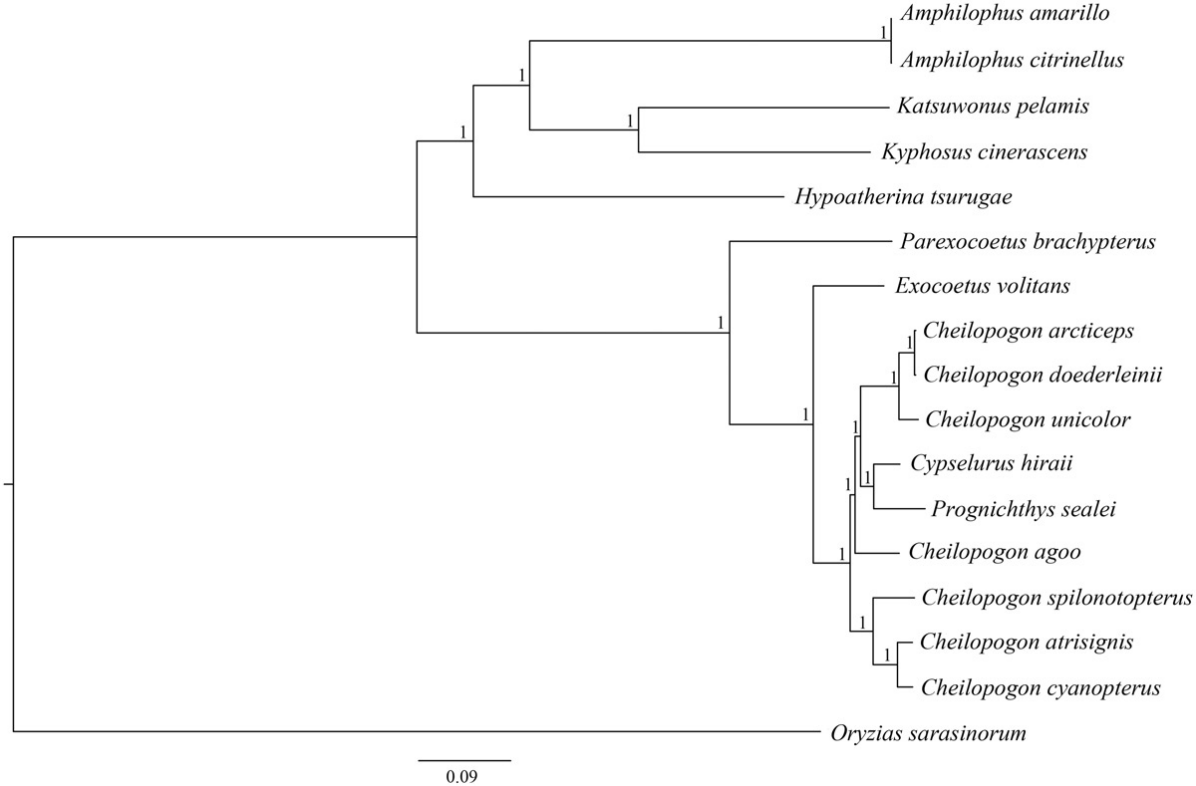
The Bayesian inference phylogenetic tree for Beloniformes based on mitochondrial PCGs and rRNAs concatenated dataset. GenBank. The gene’s accession numbers for tree construction are listed as follows: Amphilophus amarillo (KY315559), Amphilophus citrinellus (KJ562277), Katsuwonus pelamis (JN086155), Kyphosus cinerascens (AP011061), Hypoatherina tsurugae (AP004420), Parexocoetus brachypterus (KY067947), Exocoetus volitans (AP002933), Cypselurus hiraii (AB182653), Prognichthys sealei (KY067951), Oryzias sarasinorum (AB370891), Cheilopogon atrisignis (KU360729), Cheilopogon arcticeps (KU360728), Cheilopogon unicolor (KU360727), Cheilopogon doederleinii (AP017897), Cheilopogon cyanopterus (KY067950), Cheilopogon agoo (KY067949).
